# Fe-curcumin nanozyme-mediated immunosuppression and anti-inflammation in experimental autoimmune uveitis

**DOI:** 10.1186/s40824-023-00451-1

**Published:** 2023-12-12

**Authors:** Zhengxuan Jiang, Kun Liang, Xiang Gao, Fan Cao, Guangqi An, Siyu Gui, Weiwei Tang, Liping Du, Liming Tao, Xianwen Wang

**Affiliations:** 1 Department of Ophthalmology, The Second Affiliated Hospital, Anhui Medical University, Hefei, 230601 Anhui P. R. China; 2 The First Affiliated Hospital of Zhengzhou University, Academy of Medical Sciences of Zhengzhou University, Zhengzhou, 450052 Henan P. R. China; 3 School of Biomedical Engineering, Research and Engineering Center of Biomedical Materials, Anhui Medical University, Hefei, 230032 P. R. China

**Keywords:** Uveitis, Curcumin, Nanozyme, Inflammation, Immunity

## Abstract

**Background:**

EAU is an inflammatory disease usually characterized by autoinflammation and autoimmunity and is aggravated by excessive generation of ROS. Conventional hormone therapy often has more adverse effects. It is urgent to find a therapeutic drug with higher efficiency and fewer adverse effects.

**Methods:**

We developed an Fe-curcumin nanozyme in which natural antioxidants coordinate with Fe^3+^ to form nanoparticles with excellent solubility for directing anti-inflammatory and ROS scavenging effects to treat EAU. Several experiments were used to detect the characteristics of nanozymes. EAU model rats were used to detect the abilities of decreasing autoinflammation and autoimmunity. PBMCs were used to detect the ability to inhibit cell proliferation.

**Results:**

Free radical scavenging experiments showed that nanozymes decreased the level of free radicals at low concentrations. In vitro and in vivo experiments revealed that the group treated with Fe-curcumin nanozymes had lower inflammatory reactions and ROS levels than the control group, as reflected by the downregulated levels of several critical inflammatory cytokines, such as IFN-γ, IL-17, and TNF-α; decreased H_2_O_2_ release; inhibited proliferation of Th1 and Th17 cells; and alleviated pathological changes in the eye. Importantly, the Fe-curcumin nanozyme was detected in the retina using Prussian blue staining. Additionally, Fe-curcumin nanozyme is noncytotoxic when directing these biological activities.

**Conclusion:**

This study has demonstrated the feasibility of using the Fe-curcumin nanozyme as a nanodrug to inhibit inflammatory reactions and scavenge ROS in the treatment of EAU, indicating that it may serve as a promising therapeutic agent in clinical treatment.

**Graphical Abstract:**

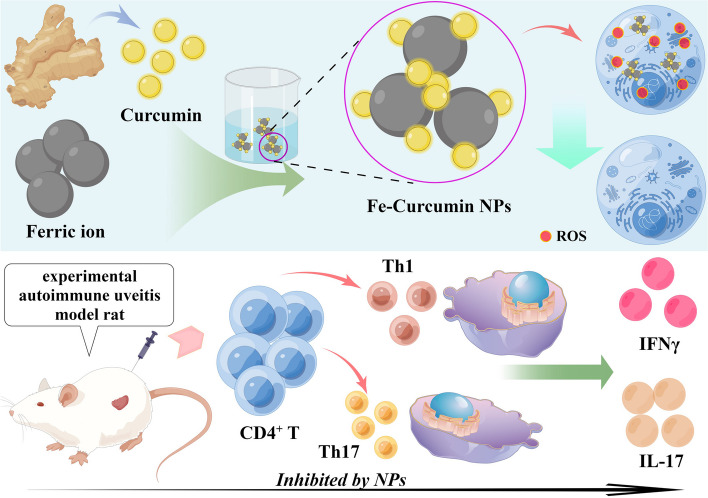

**Supplementary Information:**

The online version contains supplementary material available at 10.1186/s40824-023-00451-1.

## Introduction

Uveitis is a common eye disease that threatens sight in clinical settings. It can be divided into many types based on the mode or location of the disease [1, 2]. In particular, most noninfectious uveitis cases are associated with immunity, usually termed autoinflammatory or autoimmune disease, [3] and the other key mechanism in the progression of uveitis is oxidative stress [4–6]. However, treatment in clinics for patients is still limited to immunosuppressive therapy for a long time. Therapeutic drugs for uveitis include alkylating agents, corticosteroids and antimetabolites [7–10]. Unfortunately, nonspecific and systemic adverse effects often follow. Therefore, identifying more effective and low-side-effect treatment drugs is highly desirable. Recently, researchers have indicated that natural product-based nanomedicines have potential therapeutic effects on various inflammatory and oxidative diseases [11–13].

Natural products, such as curcumin, have been used to treat several diseases, including cancer, bacterial infections, metabolism and autoimmune disorders [14–17]. The effect might be mediated by their anti-inflammatory, antioxidant, and immunoregulatory capabilities [18]. Curcumin is a natural polyphenol compound with a molecular weight of approximately 368.38 g/mol. It is a hydrophobic molecule that is mostly insoluble in water but highly soluble in polar solvents (e.g., ethanol, methanol, and dimethyl sulfoxide) [19, 20]. However, its clinical application is limited due to its poor bioavailability [21–24].

Nanozymes have recently received considerable attention in the nanomaterial field as artificial enzymes due to their unique characteristics, such as advanced catalytic activity and excellent stability [25–30]. The term “nanozyme” was first coined in 2004, and since then, nanozymes have evolved into various types in the following decade of research [18, 31–34]. Nanozymes can be divided into two types (single-substrate mechanism or multisubstrate mechanism nanozymes) based on the number of reacted substrates [35–38]. One representative single-substrate mechanism is that of superoxide dismutase (SOD) [39]. Importantly, SOD can degrade superoxide radicals into oxygen and hydrogen peroxide [40]. Fe-curcumin nanozymes, with excellent solubility and characteristics of SOD-like enzymes, composed of natural curcumin molecules and ferric ions, were reported to cure acute kidney and acute lung injuries [41, 42]. The nanozyme perfectly combines the characteristics of curcumin and multivalent metal ions (Fe^2+/3+^).

**Scheme 1:**
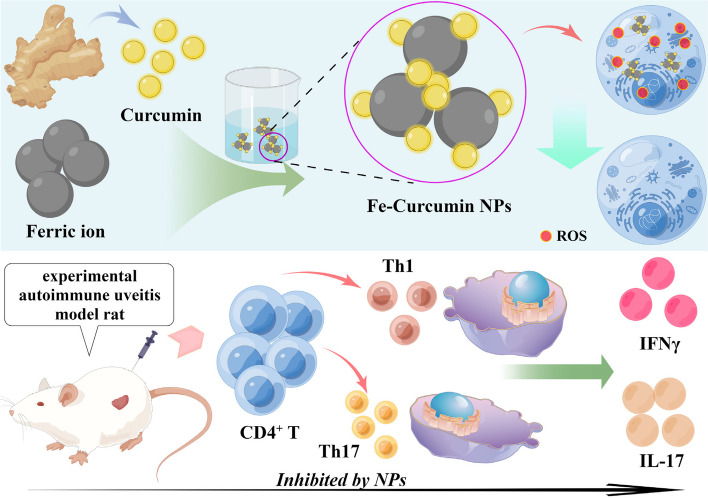
Fe-curcumin NPs were successfully prepared for experimental autoimmune uveitis therapy

In the present study, the excellent Fe-curcumin nanozyme was used to treat experimental autoimmune uveitis (EAU, a stable animal model of uveitis) for the first time (Scheme 1). We systematically detected the related inflammatory factors and oxidative stress levels and studied the mechanisms of cellular differentiation both in vitro and in vivo. It was found that Fe-curcumin nanozymes were stable owing to their lack of change in characteristics after being stored at room temperature for 7 days. Further results demonstrated that several important inflammatory cytokines, such as interferon (IFN)-γ, interleukin (IL)-17, and tumor necrosis factor (TNF)-α, could be downregulated, H_2_O_2_ levels could be decreased, and Th1 and Th17 cell differentiation could be inhibited. Additionally, drug concentrations within a certain range have no cytotoxicity. These findings suggested that the Fe-curcumin nanozyme is likely to be a valuable tool for laboratory research and clinical practice.

## Materials and methods

### Reagents and solvents

Curcumin was purchased from Shanghai Sigma-Aldrich Co., Ltd. Polyvinylpyrrolidone ([PVP], K30) and ferric chloride hexahydrate (FeCl_3_▪6H_2_O) were purchased from Macklin Biochemical Co., Ltd. (Shanghai, China). Cell Counting Kit-8 (C0037) and Antifade Mounting Medium with DAPI (P0131) were purchased from Beyotime (Shanghai, China). Dulbecco’s modified Eagle’s medium (DMEM), RF/6A cell-specific medium (CM-0194), and phosphate-buffered saline ([PBS], pH 7.4) were purchased from Wuhan Procell Life Technology Co., Ltd. (Hebei, China). Fetal bovine serum was purchased from Gibco (Gibco, Australia). Penicillin-streptomycin solution (100×), trypsin solution (EDTA-free), and trypsin-EDTA solution were purchased from Biosharp (China). Rat enzyme-linked immunosorbent assay (ELISA) kits for IFN-γ, IL-17, and TNF-α were purchased from Enzyme-linked Biotechnology Co., Ltd. (Shanghai, China), and human ELISA kits for the same three factors were purchased from Hangzhou Lianke Biotechnology Co., Ltd. (Zhejiang, China). All primary antibodies for immunofluorescence were purchased from Affinity Biosciences Biotechnology Co., Ltd. (Jiangsu, China) and Beijing Bioss Biotechnology, and all antibodies for flow cytometry were purchased from Wuhan Sanying Biotechnology Co., Ltd. (Hubei, China) and Biosciences. All antibody information is listed in Table 1. All reagents and solvents were used as obtained without further purification.

**Table 1:** All antibodies used in the experiments

Antibody	Vendor	Number
IL-17 (IF)	affinity	DF6127
TNF-a (IF)	affinity	AF7014
IFN-γ (IF)	bioss	bs-0480R
IL-17 (Flow Cyt)	wuhansanying	66148-1-Ig
IFN-γ (Flow Cyt)	bdbiosciences	559,499

### Synthesis of Fe-curcumin nanozyme

Fe-curcumin nanozymes were synthesized by a simple and well-established method [11]. First, 132 mg PVP and 40 mg FeCl_3_•6H_2_O were dissolved in 10 mL and 2 mL methanol, respectively. Subsequently, the two solvents were mixed by stirring. After 5 min, 20 mg of curcumin dissolved in 2 mL of methanol was added dropwise and stirred for 3 h. The mixed solution was dialyzed against water for approximately 12 h. Finally, the dialysis products were collected on standby after ultrafiltration.

### Characterization of the Fe-curcumin nanozyme

Images of the Fe-curcumin nanozymes were obtained using transmission electron microscopy ([TEM], Talos L120C, Thermo Scientific, USA). Dynamic light scattering ([DLS], Malvern-Zetasizer-Nano-ZS90, UK) was performed to determine the hydrodynamic sizes of the nanozyme. The organofunctional groups of the nanozyme were characterized using Fourier transform infrared (FTIR) spectroscopy (Thermo Scientific Nicolet iS20, USA). The elemental composition and valence distribution were determined using X-ray photoelectron spectroscopy ([XPS], Thermo Scientific K-Alpha, USA), and the concentration of the nanozyme was calculated in terms of Fe as measured using an inductively coupled plasma-atomic emission spectrometer (ICP-OES).

### Radical scavenging assays

First, 1,1-diphenyl-2-picrylhydrazyl (DPPH), 2,2′-azino-bis(3-ethylbenzothiazoline-6-sulfonic acid) (ABTS), and methylene blue (MB) assays as well as the determination of electron spin resonance (ESR) spectra were performed. DPPH is a stable free radical of the nitrogen center, which fades in the presence of antioxidative agents. ABTS is a free radical of oxygen, which showed a characteristic absorption peak at 734 nm. The MB test is used to describe the ability to scavenge ▪OH radicals. Second, we confirmed whether the Fe-curcumin nanozyme has the characteristics of SOD [43].

### Cell culture

Peripheral blood mononuclear cells (PBMCs) were obtained from one of the investigators of this study. Fresh human peripheral blood was centrifuged after being treated with human lymphocyte isolate, diluted to the “white part” layer and centrifuged again. PBMCs were in the precipitate. The RF/6A cell line was obtained from Wuhan Procell Life Technology Co., Ltd. All cells were cultured in a special culture medium in an incubator at 37 °C with 95% O_2_ and 5% CO_2_. All cell experiments were conducted with cells within 8 generations.

### *In vitro* cytotoxicity and reactive oxygen species (ROS) scavenging assays

PBMCs were cultured under standard conditions. For the cytotoxicity assay, PBMCs were cultured with eight concentrations of Fe-curcumin nanozyme (400, 300, 200, 100, 50, 25, 12.5, and 0 µg/mL) for 0, 12, and 24 h, and the Cell Counting Kit-8 (CCK8) assay was performed to test relative cell viability.

To protect cells from H_2_O_2_-induced oxidative stress, RF/6A cells were cultured with 1 mM H_2_O_2_ in the absence or presence of Fe-curcumin nanozyme (100 µg/mL) for 30 min. Subsequently, 100 µM fluorescence probe was added to detect intracellular ROS generation. After being washed three times with serum-free medium, the cells were observed under a laser scanning confocal microscope.

### Cell ELISA and flow cytometry

PBMCs (4 × 10^5^ cells/well) were cultured in 6-well plates for approximately 24 h. Next, the cells were randomly divided into four groups: control, control with nanozyme treatment, model, and model with nanozyme treatment. Lipopolysaccharide (1 µg/mL) + IFN-γ (500 ng/mL) was used to stimulate cell differentiation to simulate the EAU model at the cellular level. Fe-curcumin nanozyme at an injection dose of 100 µg/mL was used to conduct this experiment. After another 24 h of culture, PBMCs were collected and completely lysed in lysis buffer under oscillation. The supernatant was collected and analyzed using ELISA. Flow cytometry, which was performed by Hefei DingXiu Biotechnology Co., Ltd., was used to determine the differentiation levels of Th1 and Th17 cells.

### Animal model and treatments

Adult 7-week-old male Lewis rats (*n* = 20) were provided by Beijing Charles River Laboratories Animal Co., Ltd. (Hunan, China). All animals were given *ad libitum* access to food and water during the acclimation week and were fed a standard diet. Animal experimental protocols were reviewed and approved by the Animal Care and Use Committee of Anhui Medical University in accordance with the International Guiding Principles for Biomedical Research Involving Animals of CIOMS (No. 20,200,503).

Two milliliters of human intercellular retinol-binding protein (IRBP_161 − 180_, 1 mg/mL) and 2 mL of complete Freund’s adjuvant were completely blended in a three-way pipe for 2 h until a milky liquid was obtained. Rats received a double rear footpad injection of IRBP (200 µL, 100 µL each) and intraperitoneal injection of pertussis toxin (PTX, 100 µL) for the EAU model. Rats were divided into four groups: control, control + Fe-curcumin NP, EAU, and EAU + Fe-curcumin NP. The control and EAU groups received normal saline during the experiment, whereas the corresponding treatment groups received nanoparticle solution for 11 consecutive days. Additionally, 5 µL of Fe-curcumin nanozyme was injected into the eye through the vitreous cavity on the 7th day of the experiment. At the peak time of inflammation, i.e., on the 14th day, the rats were sacrificed for subsequent experiments.

### *In vivo* detection methods

After fasting, the rats were anesthetized with 10% pentobarbital sodium and euthanized via cervical dislocation at the end of the experiment. Owing to the scarcity of experimental rat eyes, it was necessary to use the tissues in a reasonable manner. Half of the tissue was used for pathological examination (H&E staining, immunofluorescence, and Prussian blue staining), while the other half was completely homogenized to obtain the supernatant for ELISA. Whole blood was collected from the orbital vein and stored at a low temperature (4 °C) for 12 h. Following clear stratification of serum and plasma, the serum was separated via centrifugation for ELISA and H_2_O_2_ assays. The spleens of the animals were isolated and preserved in normal saline for flow cytometry.

### *In vivo* flow cytometry

Splenic lymphocyte separation: Red blood cell lysate was added after grinding the rat spleen, and the spleen was washed twice with PBS. Cell stimulation activation: Activating solution was added to lymphocyte precipitation and resuspended. Antibody staining: cell surface antigen labeled with flow cytometry buffer and CD4 antibody; polyaldehyde, membrane breaker and corresponding antibody label cell internal antigen.

### Toxicity of the Fe-curcumin nanozyme

The weight of all rats was monitored and recorded every other day. Major organs (heart, kidney, and brain) were collected on day 14 at the end of the experiment, and the body weight was recorded every 2 days. The biosafety of the Fe-curcumin nanozyme was evaluated in all treatment groups. Additionally, H&E staining was performed as a visual and quantitative index to evaluate biosafety.

### Statistical analysis

Quantitative experimental data, such as ELISA, H_2_O_2_ assay, and body weight measurements, were analyzed using SPSS 22.0 software (IBM Corporation, USA) and are expressed as the means ± standard deviations in graphs. One-way analysis of variance was used to compare the in vivo and in vitro experimental data among all four groups. A *t* test was used to compare the results between the two groups. A *p* value of ≤ 0.05 was considered statistically significant. All graphics, including bar and line charts, were drawn using Origin 2021 and GraphPad Prism 8.0.1.

## Results

### Characterization of the Fe-curcumin nanozyme

**Fig. 1:**
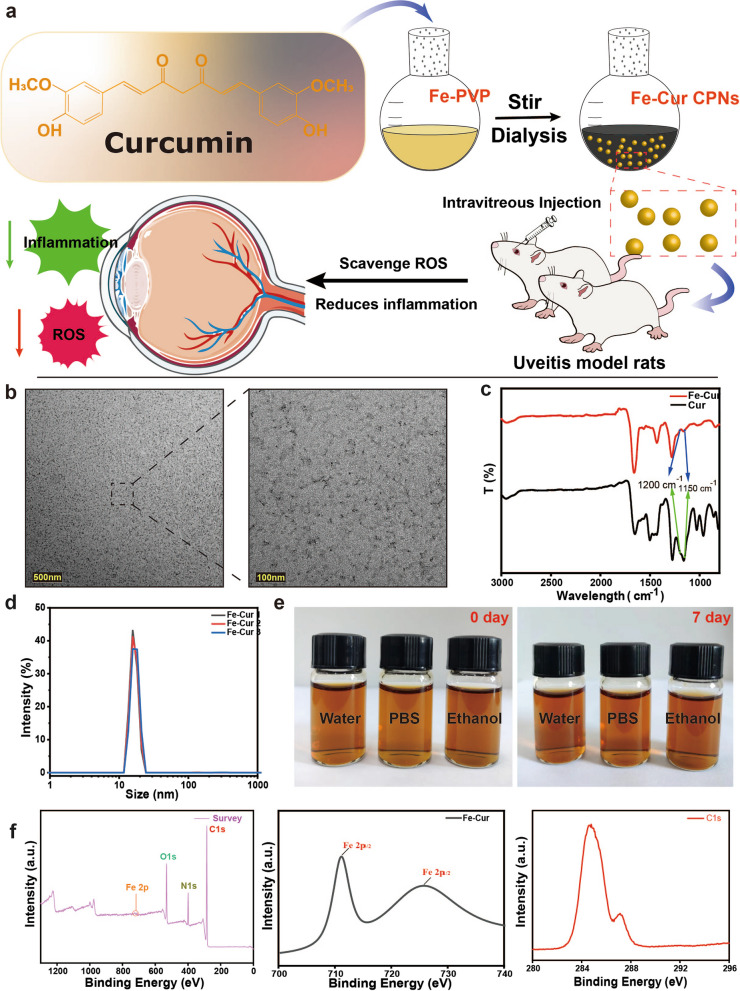
Characterization of the Fe-curcumin nanozyme. **a** The preparation process of the Fe-curcumin nanozyme. **b** TEM image. The picture was snapped by 100 µg/mL Fe-curcumin ethanol solution. **c** FTIR spectra of curcumin and Fe-curcumin samples. **d** DLS data of the Fe-curcumin nanozyme. **e** Photos of the Fe-curcumin nanozyme in different solvents. **f** XPS spectra of the Fe-curcumin nanozyme. Fe and C1s are provided separately

The Fe-curcumin nanozyme was synthesized via a simple method in which natural curcumin powder was added to a mixed solution (FeCl_3_▪6H_2_O and PVP dispersed in methanol), wherein PVP was used to improve the solubility and stability of the nanoparticles in water (Fig. 1a). TEM was performed to observe the morphology and microstructure of the sample. The size of the Fe-curcumin nanozyme was approximately 5 nm in the TEM images (Fig. 1b). Meanwhile, it was purified and stored in reserve after dialysis and ultrafiltration. To confirm the accuracy of coordination between curcumin and ferric ions, FTIR was used to test the infrared intensity of the Fe-curcumin nanozyme and natural curcumin (Fig. 1c). The results revealed that Fe^3+^ coordinated with curcumin, as a downward trend was observed (at 1150–1200 cm^−1^, which corresponds to the HO-C stretching band). Finally, the hydrodynamic sizes of the nanozyme were measured using DLS, and the sizes appeared slightly larger than those observed under TEM **(**Fig. 1d), which may be because of agglutination of the nanozyme in an aqueous solution. Additionally, similar to the findings of a previous study, after Fe^3+^ was coordinated with the phenol groups of curcumin, the yellow curcumin solution turned into a black Fe-curcumin solution. The Fe-curcumin nanozyme was dissolved in three solvents: water, PBS, and ethanol. The UV-vis absorption wavelength of the particles showed that there was no change at 0 and 7 days. Meanwhile, a short-term experiment has shown that drugs are more suitable for alkaline environments (Fig. 1e and Figure S1). XPS analysis was performed for further characterization. The results suggested that the nanozyme was mainly composed of Fe 2p, O 1s, N 1s, and C 1s (Fig. 1f, left). Two strong binding energy peaks at 711 eV and 724 eV were observed for Fe 2p_3/2_ and Fe 2p_1/2_ of Fe^3+^, respectively, indicating that ferric ions maintain their inherent oxidant ability in the new nanoparticles (Fig. 1f, middle).^[42]^ Additionally, the peak deconvolution results for C 1s are indicated on the right side of Fig. 1f. The ratio of Fe in the Fe-curcumin nanozyme was determined using ICP-OES. The results showed that the weight of Fe was approximately 252.6 µg in 1000 µg Fe-curcumin nanozyme. These results proved that the synthesis of the Fe-curcumin nanozyme was successful.

### Various radical scavenging abilities of the Fe-curcumin nanozyme

**Fig. 2:**
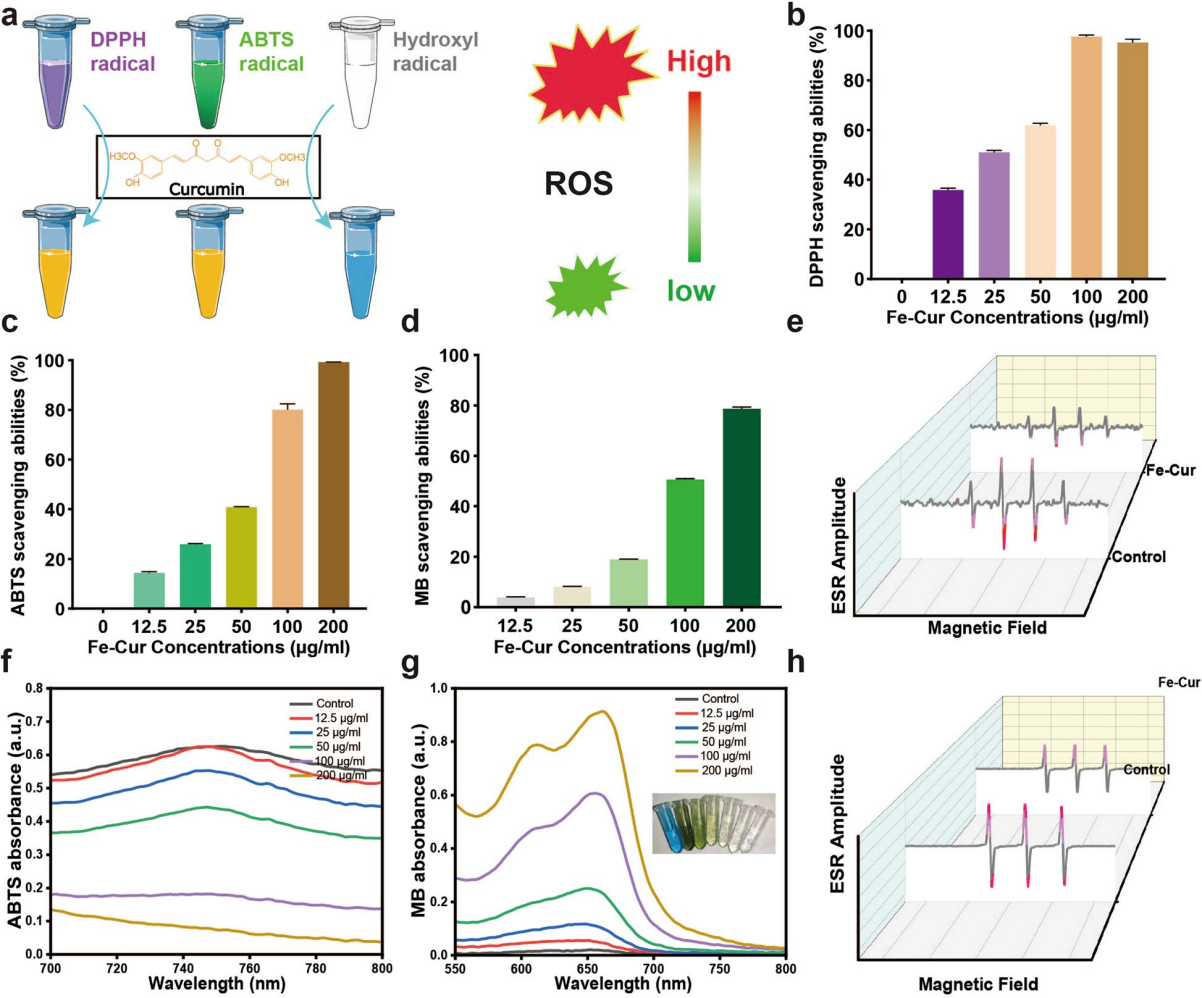
ROS scavenging ability of the Fe-curcumin nanozyme. **a** Schematic illustration of the ROS scavenging process. **b** DPPH radical scavenging ratio of the Fe-curcumin nanozyme. **c** ABTS radical scavenging ratio of the Fe-curcumin nanozyme. **d** Data statistics of the ROS scavenging ability based on the UV-vis absorption spectra of MB. **e** ESR spectra of DMPO indicating ▪OH capture with or without the Fe-curcumin nanozyme. **f** UV-vis absorption spectra of ABTS with different treatments. **g** UV-vis absorption spectra of MB with different treatments. **h** ESR spectra of TEMPO indicating singlet oxygen with or without the Fe-curcumin nanozyme

We carefully verified the antioxidative ability in the Fe-curcumin nanozyme **(**Fig. 2a**)**. Four different methods were used to perform the experiment. First, DPPH was used to evaluate the radical scavenging ability of the nanozyme (Fig. 2b). DPPH radicals appear purple and represent a significant absorption peak at 517 nm. As a free radical model in many radical scavenging tests, the characteristic peak of DPPH gradually decreased, and the purple color slowly faded with increasing concentrations of antioxidative agents [44]. Interestingly, we found that the Fe-curcumin nanozyme showed strong scavenging ability in the DPPH test. The purple color disappeared under 50 µg/mL nanozyme treatment, and 100 µg/mL nanozyme exhibited the highest scavenging ability. Additionally, another method, the ABTS assay, was used to further verify the radical scavenging ability of the nanoparticles [45]. Fig. 2 shows that DPPH radicals decreased by more than 90%, but ABTS radicals decreased by approximately 80% under 100 µg/mL Fe-curcumin nanozyme treatment (Fig. 2b-c, f). When the nanozyme was added at a concentration of 200 µg/mL, ABTS radicals decreased by more than 90%, but DPPH scavenging no longer increased. Meanwhile, some radicals, particularly ▪OH radicals, can accelerate inflammatory and oxidative progression in various injuries and diseases. MB is blue and can be degraded to a colorless form in the presence of ▪OH. In this experiment, ▪OH radicals were generated via the Fenton reaction (H_2_O_2_ can be resolved to ▪OH under Fe^2+^), following which the addition of Fe-curcumin nanozyme can prevent the blue color from fading despite the generation of ▪OH. With increasing Fe-curcumin nanozyme concentration, the fading of MB decreased gradually, indicating the excellent scavenging ability of the Fe-curcumin nanozyme against ▪OH radicals (Fig. 2d, g). The chemical reaction equations for the radical scavenging process are provided in Figure S4. Moreover, ESR spectra can also be used to detect ▪OH radicals generated via the Fenton reaction. Two radical trapping agents, 5,5-dimethyl-1-pyrroline N-oxide (DMPO) and 2,2,6,6-tetramethylpiperidine-1-oxyl (TEMPO), were used in this experiment. DMPO was used to trap ▪OH radicals in ESR, whereas single oxygen was usually trapped using TEMPO (Fig. 2e, h). The results revealed that the ESR amplitude decreased when the Fe-curcumin nanozyme was added in the two ESR experiments. The SOD enzyme test shows that there is a similar-enzyme effect in the Fe-curcumin nanozyme, indicating that nanozyme catalysis of superoxide anion radical disproportionation to produce oxygen and hydrogen peroxide is possible. Similarly, the glutathione peroxidase (GPx) assay also demonstrated that the scavenging of peroxide by the Fe-curcumin nanozyme acts similar to GPx (Figure S2). Some studies have compared the efficacy of various nanoenzymes in scavenging free radicals. Furthermore, we compared three kinds of nanoparticles for scavenging active oxygen and identified the advantages of Fe-curcumin (Figure S5). In summary, the Fe-curcumin nanozyme achieved a good effect in the abovementioned tests as an ROS or radical scavenging agent.

### *In vitro* cellular experiments of the Fe-curcumin nanozyme

**Fig. 3:**
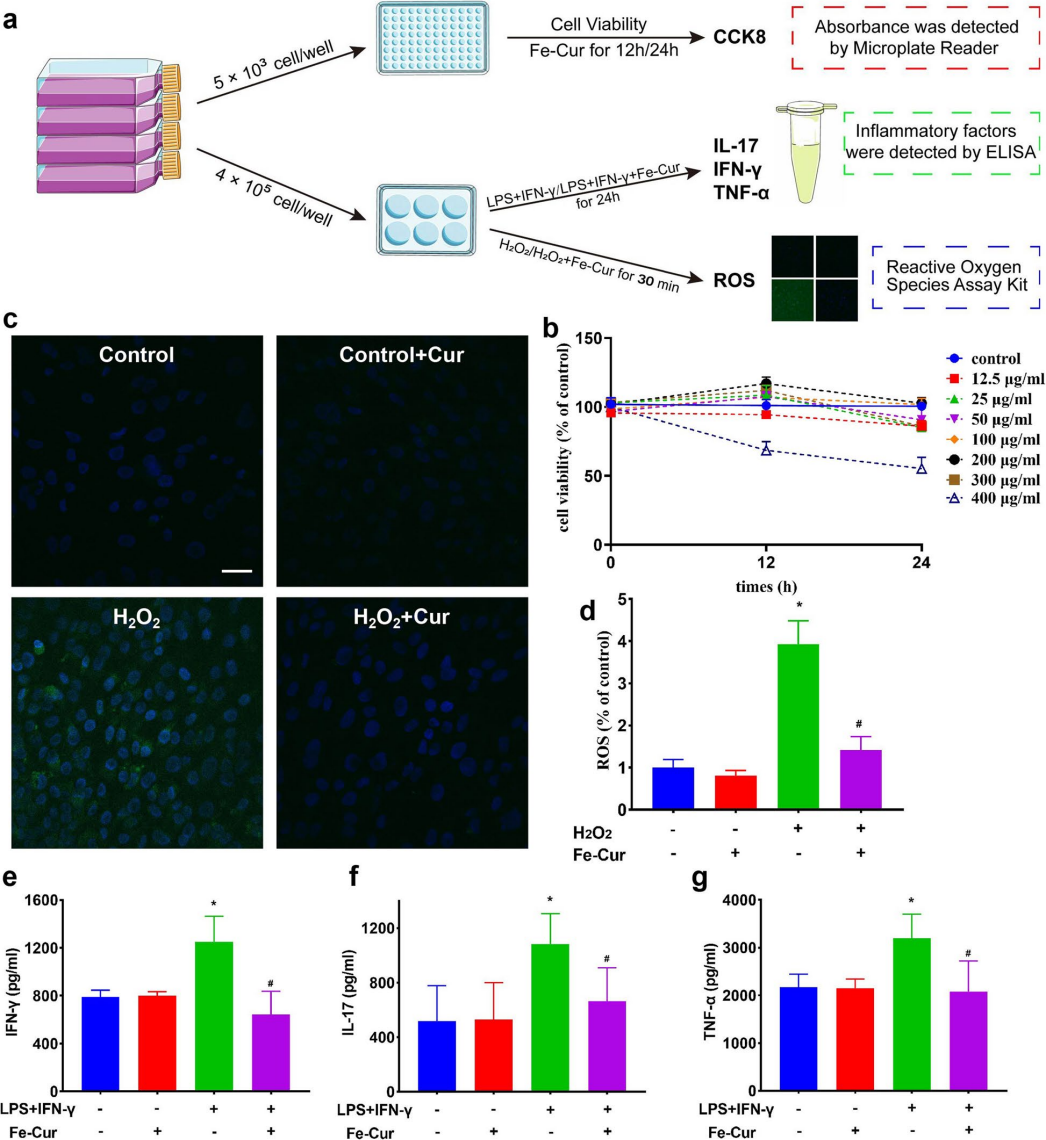
In vitro cellular experiments of EAU therapy by the Fe-curcumin nanozyme. **a** Schematic illustration of the experiments. **b** Cell viability of PBMCs was determined via CCK8 assays. The experiment screened the most suitable drug concentration for cell experiments. **c**, **d** Laser scanning confocal microscopy images of DFCH-DA-stained RF/6A cells and strength analysis after different treatments. **e, g**. ^*^ *P* < 0.05 compared with the control group; ^#^ *P* < 0.05 compared with the LPS + IFN-γ alone group

To further investigate the antioxidative and anti-inflammatory effects of the Fe-curcumin nanozyme in cells, we performed the following experiments (Fig. 3a). The cytotoxicity of the Fe-curcumin nanozyme was determined via CCK8 assay. The cell viability was the highest at a nanozyme concentration of 100 µg/mL and a culture time of 24 h (Fig. 3b). Subsequently, the ROS scavenging effect of the Fe-curcumin nanozyme was further confirmed in H_2_O_2_-treated RF/6A cells. After preculture with the Fe-curcumin nanozyme for 24 h, 1 mM H_2_O_2_ was added to the cells to stimulate ROS production for 30 min. The cells were stained with DCFH-DA, and the ROS showed green fluorescence under a laser scanning confocal microscope. As shown in Fig. 3c-d, the fluorescence intensity substantially decreased in the H_2_O_2_ + Cur group compared with the H_2_O_2_ group. The positive results clearly show that the Fe-curcumin nanozyme can eliminate ROS at the cellular level. Third, PBMCs were separated from the venous blood of patients with EAU and the investigators of this study. PBMCs from patients with EAU were treated with 100 µg/mL Fe-curcumin nanozyme for 24 h, and the differentiation of Th1 and Th17 cells was detected using flow cytometry. Consistently, the proliferation of Th1 and Th17 cells was found to be decreased (Figure S3). PBMCs from the investigators were treated with LPS + IFN-γ or Fe-curcumin nanozymes. The levels of several inflammatory cytokines closely involved in EAU, such as IFN-γ, IL-17, and TNF-α, were determined. [46–48] As shown in Fig. 3e-g, stimulation with LPS + IFN-γ increased the levels of these inflammatory factors in PBMCs, whereas treatment with the Fe-curcumin nanozyme decreased these levels. Moreover, in the absence of LPS + IFN-γ, the levels of these factors were similar regardless of whether nanozymes were present.

### *In vivo* animal experiments of the Fe-curcumin nanozyme used for treating EAU rats

**Fig. 4:**
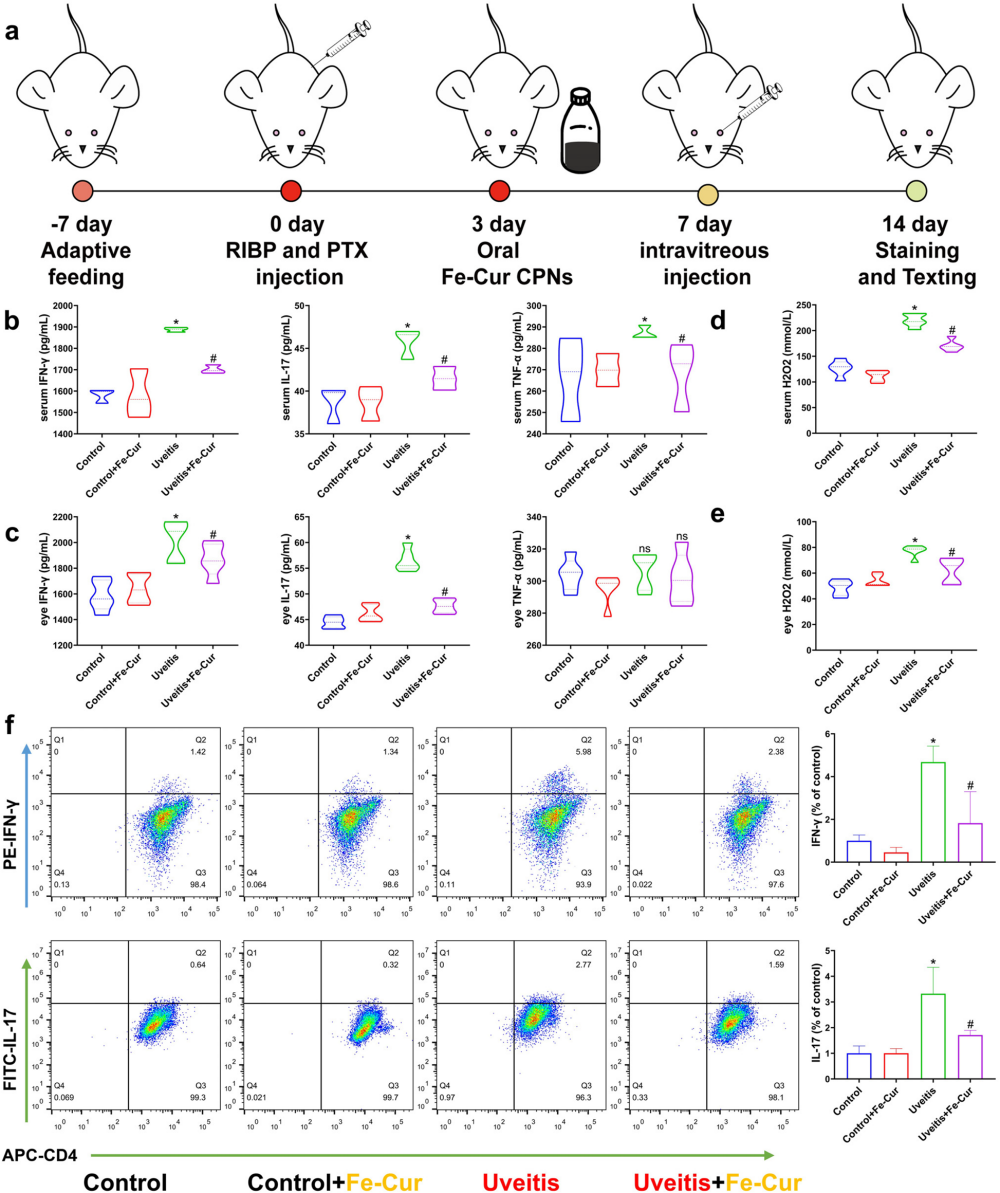
In vivo EAU alleviation with the Fe-curcumin nanozyme. **a** Schematic illustration of the experimental process. **b, c** IFN-γ, IL-17, and TNF-α levels were detected using ELISA in serum and eye tissue (*n* = 5). **d**, **e** H_2_O_2_ levels were detected using ELISA in serum and eye tissue. **f** Representative plots of Th1 and Th17 cells as a percentage of the total CD4^+^ cell population (*n* = 3)

**Fig. 5:**
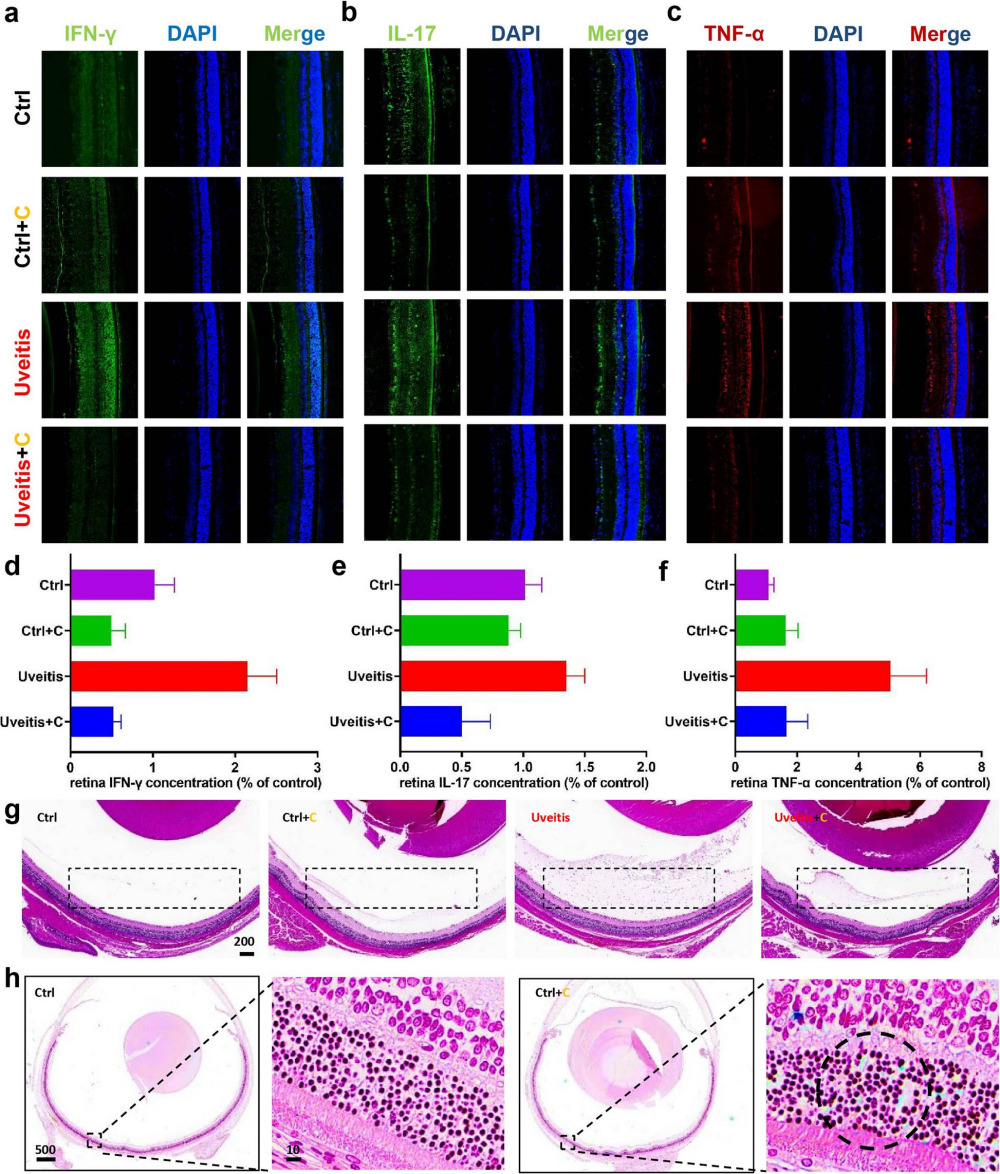
Treatment of mice with EAU with the Fe-curcumin nanozyme. **a**-c Several antibody fluorescence as determined using immunofluorescence in rat retina. **d**-**f** Quantitative analysis of fluorescence intensity. **g** H&E staining results in all rat groups. The dotted line indicates the part with inflammatory factors. **h** Prussian blue-stained retinal slices with or without Fe-curcumin nanozyme injection. The dotted line indicates the presence of stained iron ions

EAU is a potentially blinding and painful disease for most patients, and the particular inflammatory responses and excessive ROS levels in this disease serve as a clear direction for treatment. To investigate whether the Fe-curcumin nanozyme could alleviate inflammation and ROS in vivo, two groups of rats (control + Fe-curcumin NPs and EAU + Fe-curcumin NPs) received oral treatment with nanoparticle solution (10 mg▪kg^−1^▪day^−1^) simultaneously from the third day to the last day of the experiment. Considering it is uncertain whether the nanozymes can pass through the blood-retinal barrier, intravitreal injection (4 µL/eye) was performed on the 7th day of the experiment (Fig. 4a). Similarly, we further evaluated the therapeutic effect of the Fe-curcumin nanozyme in an IRBP/CFA-PTX-induced EAU animal model. After sacrificing the rats, tissues and serum were collected for detection. Inflammatory cytokines, including IFN-γ, IL-17, TNF-α, and H_2_O_2_ were detected using ELISA in the eye and serum. Fresh spleen tissue was preserved for flow cytometry to assess the differentiation of Th1 and Th17 cells. The results revealed that the levels of all three inflammatory cytokines and H_2_O_2_ were abnormally increased in the EAU model and that treatment with the Fe-curcumin nanozyme significantly decreased these levels, except for TNF-α (Fig. 4b-e). The flow cytometry results were completely consistent with our expectations. The proliferation of Th1 and Th17 cells in the spleen of EAU rats was increased, and treatment with the nanozyme inhibited this proliferation. Moreover, immunofluorescence staining of retinal tissue was used to determine the distribution and intensity of inflammatory cytokines. As shown in Fig. 5a-f, fluorescence intensity represents the protein concentration, and IFN-γ, IL-17, and TNF-α levels in the EAU groups were increased compared with those in the control groups but decreased with Fe-curcumin nanozyme treatment. Furthermore, after careful research and measurement, three inflammatory cytokines were found to be secreted in different cell layers. The IFN-γ protein is secreted in the outer nuclear layer, which contains cone and rod cells. The proteins IL-17 and TNF-α are secreted in the inner nuclear layer, which contains bipolar, horizontal, and amacrine cells and the cell bodies of Müller cells. Additionally, H&E staining experiments were performed to show that the IRBP/CFA-PTX-induced EAU rat model indeed exhibited infiltration of inflammatory cells (Fig. 5g). Finally, to prove that the Fe-curcumin nanozyme was successfully located in the eye, Prussian blue staining was used to bind with Fe^3+^ of the Fe-curcumin nanozyme. Consequently, the compound was blue in appearance. We successfully located the nanozyme in the outer nuclear layer (Fig. 5h).

### Toxicity of the Fe-curcumin nanozyme *in vivo*

**Fig. 6:**
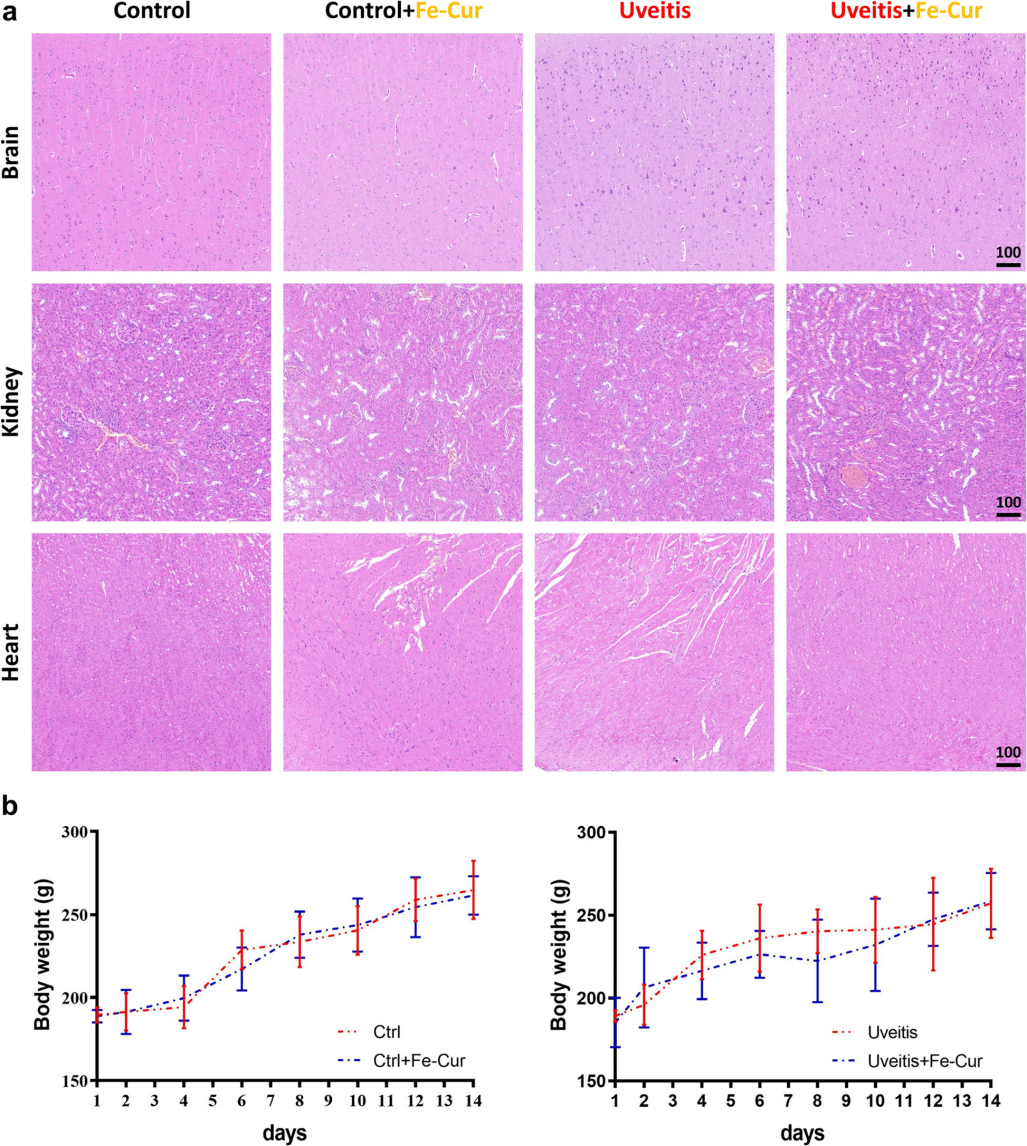
Toxicity of the Fe-curcumin nanozyme in vivo. **a** H&E staining of the brain, heart, and kidney after Fe-curcumin nanozyme treatment. There was no obvious change between groups. **b** Body weight of all mice at different time points (2, 4, 6, 8, 10, 12, and 14 days)

Most importantly, the possible in vivo toxicity of the Fe-curcumin nanozyme was evaluated. H&E staining revealed no inflammation or abnormalities in groups treated with or without the Fe-curcumin nanozymes (Fig. 6a). The body weight of rats showed no difference between the groups with or without nanozyme (Fig. 6b).

## Discussion

In this study, we report a significant alleviation of EAU by the Fe-curcumin Nanozymes. At present, there is no unified understanding or standard for the clinical treatment of uveitis worldwide. What regimen can improve the prognosis is also the focus of ophthalmologists in China. In previous clinical treatment, eye drops, although they had some effect, had a limited effect on improving the inflammatory response. With current clinical experience, glucocorticoids, immunosuppressants, interferon and so on are often used to contain the progression of inflammation. However, these therapies also have many side effects. Many inflammatory disorders are always accompanied by oxidative stress, including EAU. Meanwhile, excessive ROS can lead to protein disruption, proinflammatory cytokine expression, inflammatory infiltration, organelle damage, and cell death. [49–51] Thus, reducing inflammation together with scavenging ROS could be a potential therapeutic strategy for EAU.

With the rapid development and extensive research of nanomedicines, we tried to use nanozymes to treat uveitis. First, the biosafety of a nanozyme is critical in developing it for the treatment of any disease. Our CCK8 test indicates a nontoxic nature at a low concentration of the Fe-curcumin nanozyme. After system testing, our in vitro results demonstrate that the Fe-curcumin nanozyme has anti-inflammatory and antioxidant effects, with the mechanism primarily involving the inhibition of Th1 and Th17 cell differentiation and the secretion of IFN-γ, IL-17, and TNF-α. To further demonstrate the ability of the Fe-curcumin nanozyme, the namozyme was used to treat EAU model rats. Similar to the in vitro experimental results,  nanozymes significantly reduced the levels of inflammatory factors in the serum and eye. It is worth mentioning that TNF-α levels showed no significant difference in eye. This result may demonstrate that IFN-γ and IL-17 are more direct factors leading to EAU than TNF-α. Regarding the current results, except safety, the anti-inflammatory and antioxidant abilities of the Fe-curcumin nanozyme were confirmed entirely in vitro and in vivo. The therapeutic effect of the Fe-curcumin nanozyme in EAU is also worthy of affirmation.

However, this study had limitations that might bias our conclusions. First, we did not use the spleen of the animals as samples for a series of direct tests, which may provide new data. Second, further exploration of the molecular mechanism is not perfect, and further research is needed to determine which way Fe-curcumin nanozymes affect the secretion of inflammatory factors.

## Conclusions

In summary, Fe-curcumin nanozyme, composed of curcumin and ferric ions, was synthesized using a well-established and easy method. After confirming the anti-inflammatory and antioxidant abilities of the nanozyme, it was used to treat EAU. To assess the effect of the nanoparticles of this nanozyme, we confirmed the changes in inflammatory factor levels through various methods. Fe-curcumin nanozymes decreased ROS production in vivo and in vitro. The levels of inflammatory factors (IFN-γ, IL-17, and TNF-α) were also decreased after Fe-curcumin nanozyme treatment. Moreover, limited proliferation of Th1 and Th17 cells was observed through flow cytometry. Hence, the findings of this study may aid in the development of clinical treatment strategies for EAU.

### Supplementary Information

**Additional file 1: Figure S1.** The stability of Fe-curcumin in different condition. (a) Fe-curcumin nanozyme dispersed in water. (b) Fe-curcumin nanozyme dispersed in ethanol. (c) The first day of Fe-curcumin nanozyme dispersed in solution with different pH. (d) The third day of Fe-curcumin nanozyme dispersed in solution with different pH. **Figure S2.** Like-bioenzyme activity of Nanozymes. (a-b) SOD enzyme activity of Fe-curcumin nanozyme. (c-d) SOD enzyme activity of four common NPs. (e) GPX enzyme activity of four common NPs. **Figure S3.** Cell differentiation in patients with EAU treated with or without Fe-curcumin nanozyme. **Figure S4.** Chemical reaction equations for the radical scavenging process. **Figure S5.** Effective of several NPs in reducing ROS

## Data Availability

Not applicable.

## Abbreviations

EAU: Experimental autoimmune uveitis
ROS: Reactive oxygen species
IFN-γ: Interferon-γ
IL-17: Interleukin-17
TNF-α: Tumor necrosis factor-α
SOD: Superoxide dismutase
TEM: Transmission electron microscopy
DLS: Dynamic light scattering
FTIR: Fourier transform infrared
XPS: X-ray photoelectron spectroscopy
DPPH: 1,1-diphenyl-2-picrylhydrazyl
ABTS: 2,2-azino-bis(3-ethylbenzothiazoline-6-sulfonic acid)
MB: Methylene blue
ESR: Electron spin resonance
PBMCs: Peripheral blood mononuclear cells
CCK8: Cell counting kit-8
