# Supplementary material for: Fe-curcumin nanozyme-mediated immunosuppression and anti-inflammation in experimental autoimmune uveitis
Source: Biomater Res. 2023 Dec 12;27:131. doi: 10.1186/s40824-023-00451-1 (PMC10717250; doi:10.1186/s40824-023-00451-1)
Supplement: Supplementary file 1 — Additional file 1: Figure S1. The stability of Fe-curcumin in different condition. (a) Fe-curcumin nanozyme dispersed in water. (b) Fe-curcumin nanozyme dispersed in ethanol. (c) The first day of Fe-curcumin nanozyme dispersed in solution with different pH. (d) The third day of Fe-curcumin nanozyme dispersed in solution with different pH. Figure S2. Like-bioenzyme activity of Nanozymes. (a-b) SOD enzyme activity of Fe-curcumin nanozyme. (c-d) SOD enzyme activity of four common NPs. (e) GPX enzyme activity of four common NPs. Figure S3. Cell differentiation in patients with EAU treated with or without Fe-curcumin nanozyme. Figure S4. Chemical reaction equations for the radical scavenging process. Figure S5. Effective of several NPs in reducing ROS [file 40824_2023_451_MOESM1_ESM.doc]

Supporting Information

**Fe-curcumin Nanozyme-Mediated Immunosuppression and Anti-inflammation in Experimental Autoimmune Uveitis**

Zhengxuan Jiang^1, #^, Xiang Gao^1, #^, Kun Liang^1, #^, Fan Cao^1^, Guangqi An^3^, Siyu Gui^1^, Weiwei Tang^1^, Liping Du^3^, *, Liming Tao^1^, *, Xianwen Wang^2^, *

^1^Department of Ophthalmology, The Second Affiliated Hospital, Anhui Medical University, Hefei, Anhui, 230601, P. R. China

^2^School of Biomedical Engineering, Research and Engineering Center of Biomedical Materials, Anhui Medical University, Hefei 230032, P. R. China

^3^The First Affiliated Hospital of Zhengzhou University, Academy of Medical Sciences of Zhengzhou University, Zhengzhou, Henan 450052, P. R. China

***Corresponding Author:**

1. **mail:** [xianwenwang@ahmu.edu.cn](mailto:xianwenwang@ahmu.edu.cn) (X Wang); [taoliming@ahmu.edu.cn](mailto:taoliming@ahmu.edu.cn) (L Tao); [dulplab@live.cn](mailto:dulplab@live.cn) (L Du)

^#^ These authors contributed equally to this work.


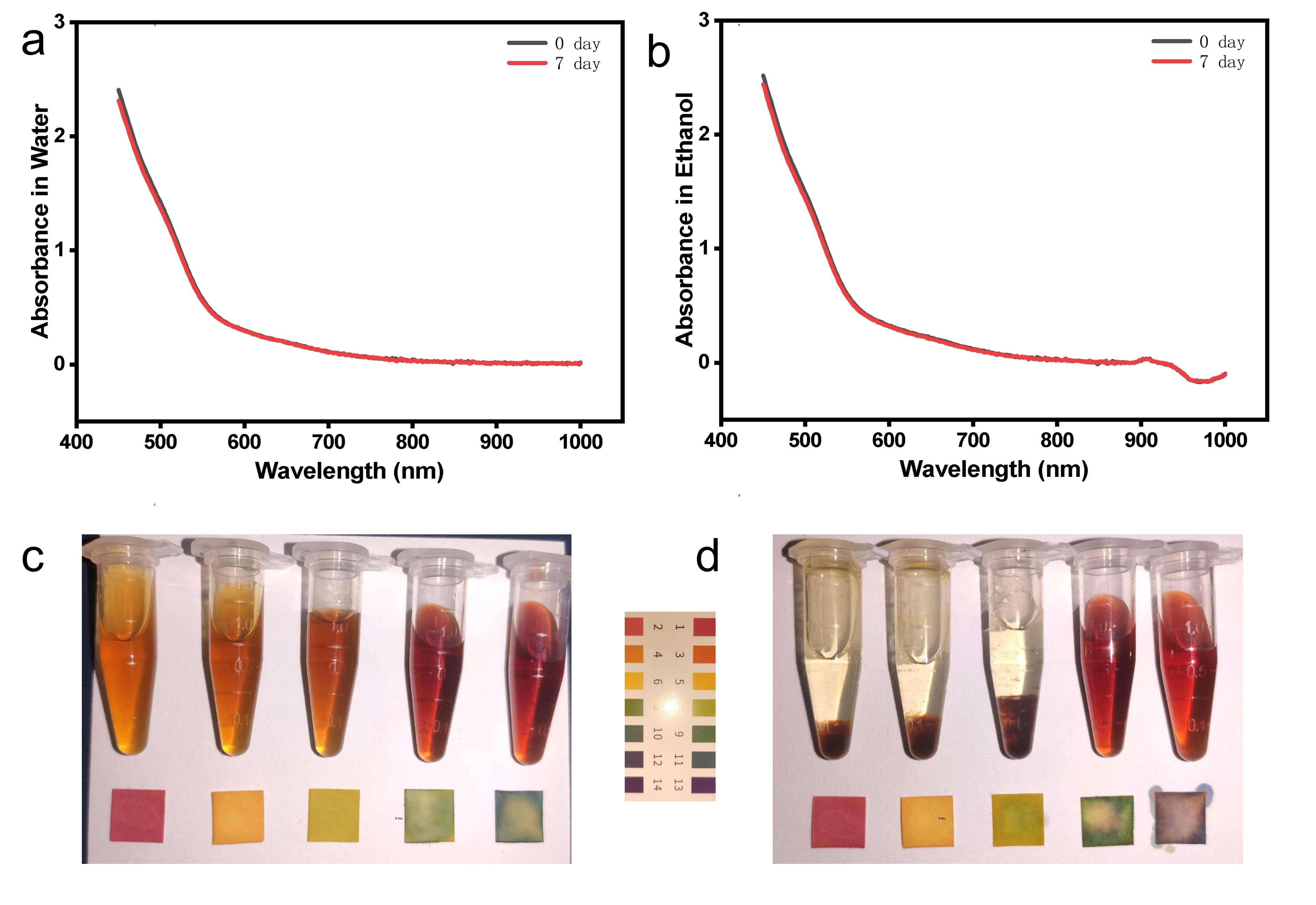


**Figure S1. The stability of Fe-curcumin in different condition.** (a) Fe-curcumin nanozyme dispersed in water. (b) Fe-curcumin nanozyme dispersed in ethanol. (c) The first day of Fe-curcumin nanozyme dispersed in solution with different pH. (d) The third day of Fe-curcumin nanozyme dispersed in solution with different pH.


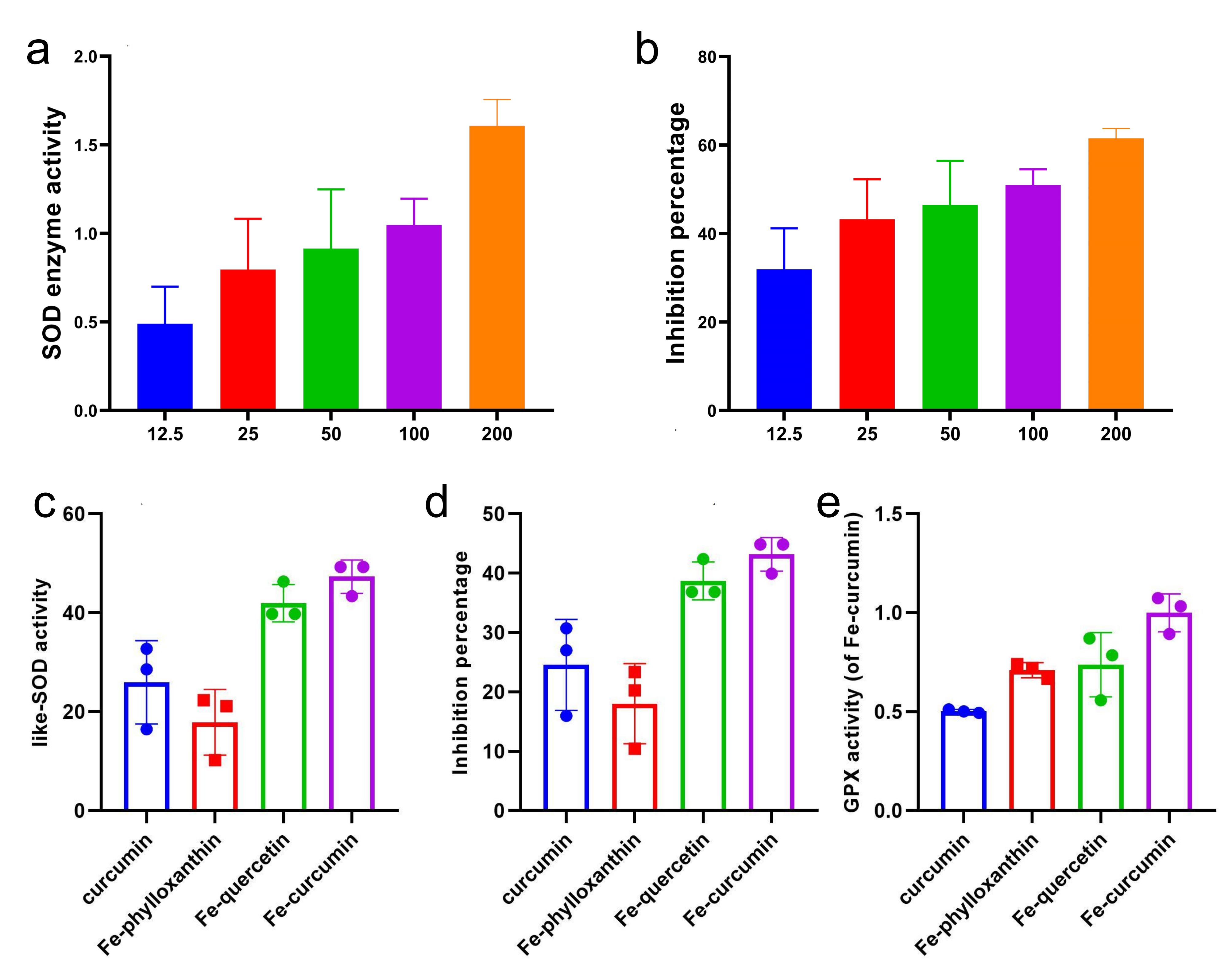


**Figure S2. Like-bioenzyme activity of Nanozymes.** (a-b) SOD enzyme activity of Fe-curcumin nanozyme. (c-d) SOD enzyme activity of four common NPs. (e) GPX enzyme activity of four common NPs.


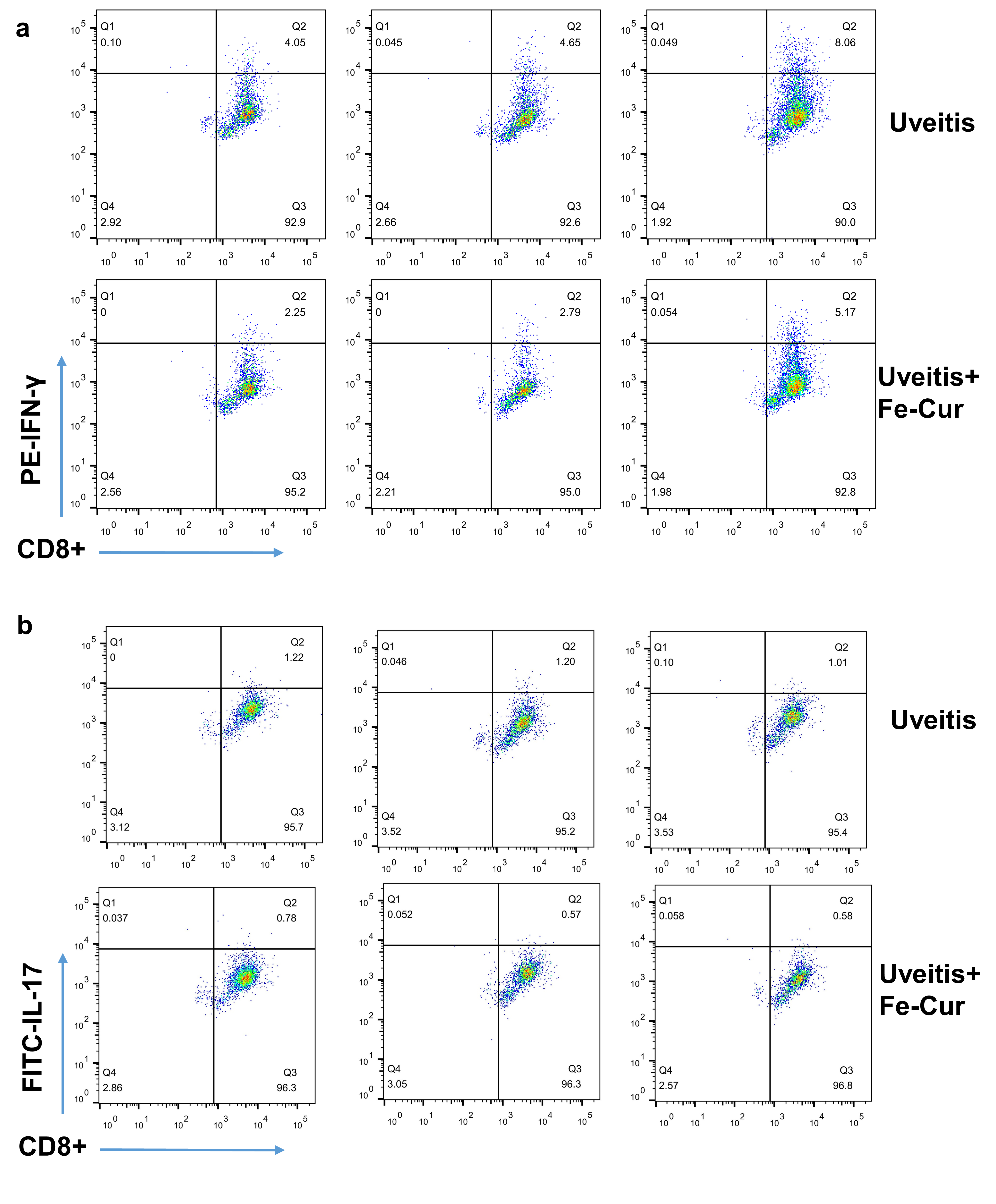


**Figure S3.** Cell differentiation in patients with EAU treated with or without Fe-curcumin nanozyme.


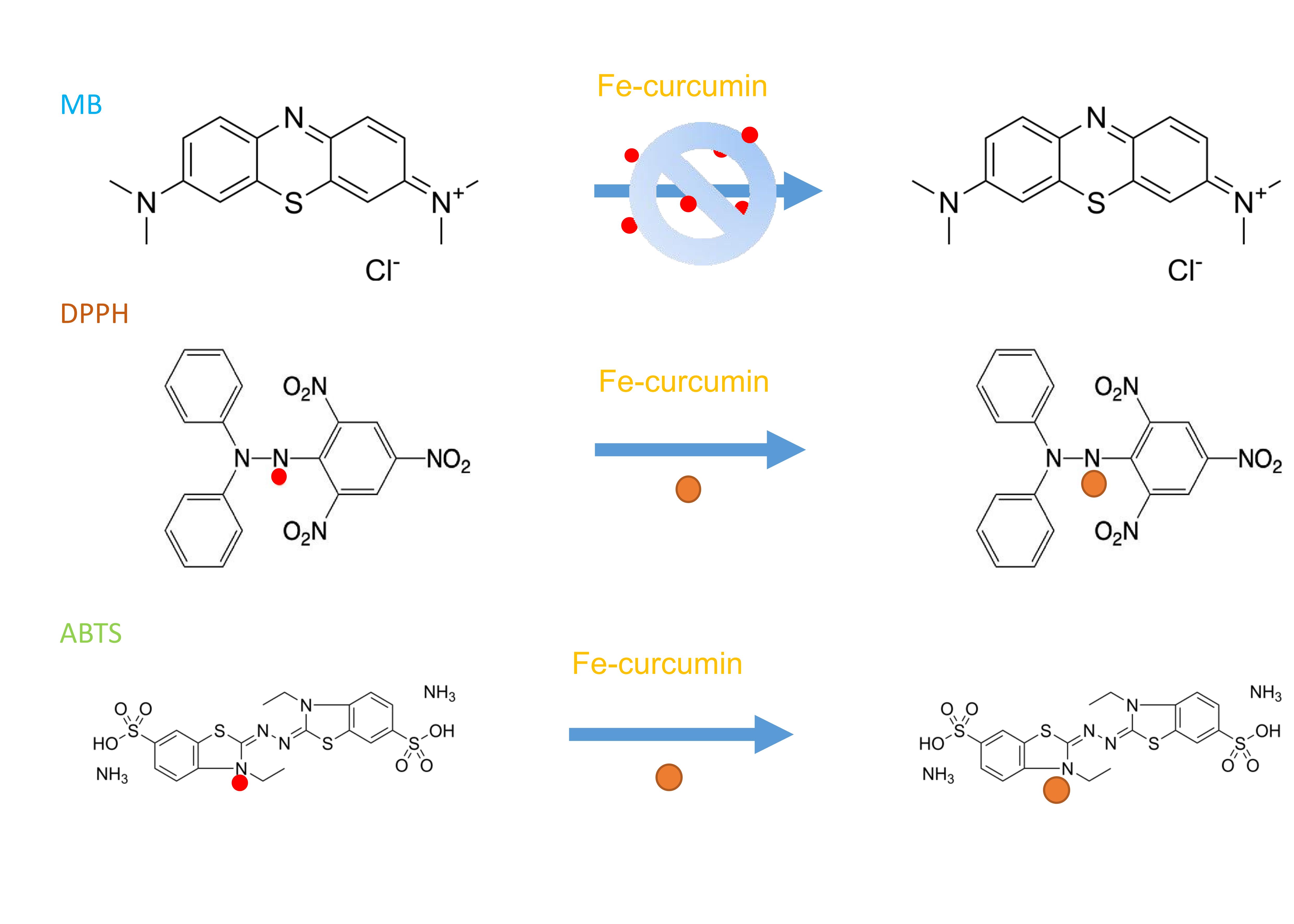


**Figure S4**. Chemical reaction equations for the radical scavenging process.


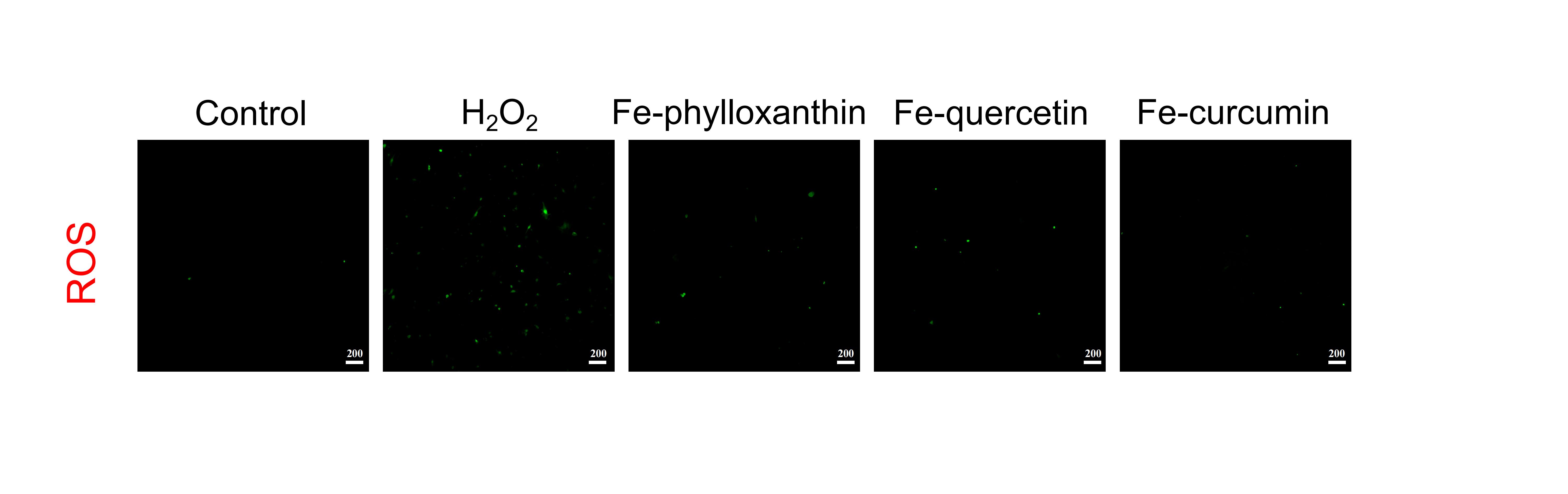


**Figure S5.** Effective of several NPs in reducing ROS.
